# Tackling the complexity of obesity in the US through adaptation of public health strategies

**DOI:** 10.3389/fpubh.2025.1477401

**Published:** 2025-04-01

**Authors:** Jamy Ard, Amber Huett-Garcia, Michele Bildner

**Affiliations:** ^1^Departments of Epidemiology & Prevention and Internal Medicine, Wake Forest University School of Medicine, Winston-Salem, NC, United States; ^2^Amber Huett-Garcia Consulting, Memphis, TN, United States; ^3^Independent Researcher, St. Louis, MO, United States

**Keywords:** equity, healthcare, obesity, policy change, public health, US

## Abstract

Obesity prevalence continues to rise in the US despite more than two decades of recommendations and guidelines for its prevention and management. The encouragement of individuals to adopt a healthy diet and lifestyle has remained the focus of clinical interventions and recommendations despite these efforts alone proving ineffective for long-term weight management. There are many recognized barriers to obesity prevention and management in community and clinical settings including political factors, social determinants of health, weight bias and stigma, and inequities in access to treatment and insurance coverage. We discuss these barriers in more detail and attempt to identify areas where public health and healthcare approaches can be better aligned, allowing for better advocating by public health officials to enable a more meaningful and population-level change in obesity prevention and management in the US.

## 1 Introduction

Obesity was first recognized as a chronic disease in a 1997 report published by the World Health Organization (WHO) and the International Obesity Task Force entitled “Obesity: Preventing and Managing the Global Epidemic” (1). The American Medical Association followed in 2013, and adopted policy recognizing obesity as a chronic disease state requiring efforts toward treatment and prevention (2), a viewpoint that has subsequently become more widely accepted (3). The Obesity Society 2018 position statement notes that obesity is covered by the classical definition of diseases as maladaptive changes from “normal” body function and structure as a result of underlying pathophysiological mechanisms, and specifically that adiposity exceeding a predefined threshold may be accompanied by structural abnormalities, functional abnormalities, signs and symptoms, elevated premature mortality risk and increased comorbidity risk (4). Public health, as defined by the Centers for Disease Control and Prevention (CDC), is “the science of protecting and improving the health of people and their communities” (5). Obesity is increasingly recognized as one of the most urgent public health issues in the US (6), affecting >40% of adults (7), with an estimated annual medical cost in the US in 2019 of $173 billion (8). The impact of obesity on public health includes effects on life expectancy, quality of life, comorbidities, employment, direct and indirect economic costs, and military readiness (6, 9). In an observational study, utilizing pooled prospective national health survey data from 114,567 adults in two Finnish cohort studies, obesity was associated with 21 non-overlapping digestive, respiratory, cardiometabolic, infectious, musculoskeletal, and neurological diseases, with a confounder-adjusted hazard ratio for developing complex comorbidity (four or more comorbid diseases) of 12.39 (95% CI 9.26–16.58; population attributable fraction 55.2% [95% CI 50.9–57.5]), compared with healthy weight. The proportion of patients with complex comorbidity was the same at age 55 years in participants with obesity as age 75 years in participants with healthy weight (10). With respect to infections (surgical site, respiratory tract, skin, and urinary tract), obesity has been identified as a risk and recurrence factor, and is often associated with more severe disease and mortality (11), particularly as noted during the COVID-19 pandemic [severe disease odds ratio [OR] 3.13, 95% CI 1.41–6.92, *p* = 0.005; mortality OR 1.36, 95% CI 1.09–1.69, *p* = 0.006; (12)]. In the US, estimates suggest that obesity accounts for more than 350,000 excess deaths per year (13), with other studies noting that an increased risk of death from all causes, and particularly from cancer and cardiovascular disease, is associated with obesity (14, 15).

Evidence suggests that ≥5% weight loss is associated with improvements in various comorbidities and associated factors including type 2 diabetes, healthcare costs, quality of life, and mortality (16). A UK study found that a median weight loss of 13% among individuals with an assumed body mass index (BMI) of 40 kg/m^2^ before weight loss (i.e., BMI 34.8 kg/m^2^ after weight loss) was associated with reductions in risk of 41% for developing type 2 diabetes (T2D), 40% for sleep apnea, 22% for hypertension, 19% for dyslipidemia, 18% for asthma, and 13% for chronic kidney disease, relative to an individual with a stable BMI of 30 kg/m^2^ (17). Guidelines by the American College of Cardiology/American Heart Association/The Obesity Society (AAC/AHA/TOS) and American Association of Clinical Endocrinologists/American College of Endocrinology (AACE/ACE) recommend multidisciplinary lifestyle interventions (e.g., aerobic exercise, resistance training, reduced-calorie diet) for the management of overweight and obesity, followed by the use of pharmacotherapy and/or surgical procedures and devices, based on factors including disease stage and success of lifestyle interventions (18–20). These interventions may include psychologists, psychiatrists, and dietitians, and should be individualized to each patient to improve adherence and outcomes. Despite these guidelines, no US state has been able to reverse the upward trends in obesity prevalence and incidence (21), suggesting that these guidelines alone are not enough to tackle the obesity epidemic, and/or that better implementation of such recommendations may be required. We know from experience gleaned during the COVID-19 pandemic, which was declared a public health emergency, that public health officials have the training and desire to quickly and effectively tackle public health crises, including facilitating primary, secondary and tertiary prevention strategies across multiple levels of influence (22, 23), yet attempts to tackle obesity remain self-evidently unsuccessful. Obesity is complex to tackle, and the state of science and will of system decision-makers differs from traditional public health initiatives such as tackling tobacco use. There are many recognized barriers to obesity prevention and management at both the public health and healthcare levels in the US, including inadequate government funding, inadequate data on effective strategies, racial disparities (24), socioeconomic factors (such as access to treatment), healthy foods (25, 26), insurance coverage (25), weight bias and stigma (27–30), and a lack of training and education of practitioners regarding effective treatment options (31).

A public health focus on prevention and treatment is needed to improve outcomes in people with overweight and obesity, as well as for addressing barriers. Effective public health campaigns in the US, such as tackling tobacco access and use to decrease and mitigate morbidity and mortality (from lung cancer and cardiovascular disease), have leveraged influence at organizational, community, societal, policy, and environmental levels. The Healthy People strategic framework, an initiative by the Office of Disease Prevention and Health Promotion, is a set of data-driven national objectives for improving health and wellbeing, using evidence-based resources to inform development of programs and policies based on what is effective, replicable, scalable, and sustainable (32). With regards to tobacco use, 13 of the 27 objectives are currently classed as “improving” or “target met” or “exceeded” in Healthy People 2030 (33). These have been driven by evidence-based strategies including smoke-free policies (34), price increases (35), health education campaigns (36, 37), counseling, and medication (38). Multi-level public-health approaches, like those implemented for smoking cessation, are key for addressing the complexity of obesity in the US. Many of these approaches are reflected in the obesity recommendations by Trust for America's Health (TFAH) for federal, state, and local policymakers and other stakeholders: (1) advance health equity by strategically dedicating federal resources to efforts that reduce obesity-related disparities and related conditions; (2) decrease food and nutrition insecurity while improving nutritional quality of available foods; (3) change the marketing and pricing strategies that lead to health disparities; (4) make physical activity and the built environment safer and more accessible for all, and; (5) work with the healthcare system to reduce disparities and close gaps in clinical-to-community settings (39).

The aim of this review is to highlight and discuss areas of obesity prevention and management in the US where public health and healthcare initiatives can be better aligned to have a more meaningful and population-level impact on the current state of obesity, and increase access to care.

## 2 Discussion

### 2.1 Diet and lifestyle interventions

Current AAC/AHA/TOS, AACE/ACE and Academy of Nutrition and Dietetics guidelines for the management of overweight and obesity recommend diet and lifestyle interventions as the first line of defense (18–20, 40). However, this achieves little sustained success, with one meta-analysis of long term maintenance of weight loss with non-surgical interventions demonstrating no evidence of effectiveness, including lifestyle changes in dietary intake and physical activity (41), and another meta-analysis of long-term weight loss studies demonstrating that the average individual only maintained a reduced weight of ~3% of initial body weight 5 years after completing a structured weight-loss program (42). This illustrates the relapsing nature of obesity as a chronic disease and highlights the need for long-term follow-up and support to help patients manage weight recurrence. Although it is clear that diet and lifestyle interventions alone are insufficient to make substantial changes in obesity outcomes broadly, most healthcare professionals (HCPs) in the ACTION study, which explored barriers to medical care and support and perspectives on obesity, agreed that general improvements in eating and physical activity are “completely effective” for long-term weight management (43). This highlights the failure among HCPs in recognizing obesity as a chronic relapsing disease that is more complex than voluntary overeating and inactivity, and further makes the case for a holistic approach to obesity management, supported by a multidisciplinary team (MDT), as well as a need to improve education among HCPs.

Patients' best efforts to incorporate recommended interventions can be counteracted or undermined by the obesogenic environment. Thus, despite decades of advice to adopt healthy eating patterns and reduce intake of added sugars, solid fats, and sodium, the Healthy Eating Index (HEI) score 2020 (an assessment of how well diets of the US population align with dietary patterns and key recommendations published by the Dietary Guidelines for Americans), was only 58/100 for ages 2+ years, demonstrating that the average diet quality does not align with the guidelines (44). This relates to changes in the food environment over the last few decades including increased food availability, which in turn has resulted in “added value” foods with altered appetitive properties that drive consumption, changes in normative eating behaviors e.g., snacking between meals (45), and increased consumption of ultra-processed foods (46). The importance of tackling the obesogenic environment is reflected in recommendations 2–4 of the TFAH obesity 2023 report (39). Various public health strategies and approaches can be undertaken by communities and neighborhoods to improve diet, behaviors, and physical activity in line with these recommendations, including advocating for zoning that limits fast food establishments, increasing the availability of farmers' markets, facilitating the situation of supermarkets in underserved areas, enhancing walking infrastructure, and increasing the number of parks and bike paths (47). Produce prescription projects are also becoming increasingly common, whereby HCPs can “prescribe” fruit and vegetables for patients experiencing food insecurity. With respect to aligning this with public health, suggestions by the CDC include engaging representatives from Medicaid programs in implementing, expanding and evaluating produce prescription programs, and supporting policies that increase participation in prescription initiatives (48).

Addressing the obesogenic environment and instituting effective strategies for tackling obesity is a complex challenge. There is mixed evidence on the effectiveness of traditional public health approaches such as introducing supermarkets in food deserts and improving access to healthier food options on nutritional inequality, dietary habits, or obesity (49–52). Alternative measures for obesity prevention have been instigated or investigated in other countries around the world, albeit again with mixed results. At least one type of sugar-sweetened beverage (SSB) tax is enforced by more than 100 countries worldwide (53) but the impact is varied, with one systematic review finding a positive effect of this tax on decreasing the prevalence of overweight and obesity (54), however another study found no significant change in consumption of taxed beverages (55). Calorie labeling on menus is another measure that has been projected to prevent cases of obesity across all racial and ethnic groups [126–185 cases per 100,000 people; (56)]. However, a review of studies assessing the real-world impact of numeric calorie posting found the evidence to be mixed, and the effect of menu labeling to be dependent on context (57). For example, low-income customers may respond to labeling by choosing higher-calorie options, as they perceive items containing more calories per dollar as being of better value (58).

These measures are also sometimes considered controversial, with issues including resistance lobbying and legal action, such as in the case of the New York SSB portion cap being overturned (59). The prospect of resistance makes it more difficult to build stakeholder support.

Another aspect of SSB taxes that makes them controversial is that they can be considered a “regressive tax.” In the US, households in the lowest income quintile spend ~31% of their income on food, which is nearly 4 times the amount of households in the highest income quintile [~8%; (60)]. Therefore, SSB taxes have the greatest impact on those with lower incomes, particularly if the tax results in a substantial price pass-through to the consumer, as was observed in Portugal following the introduction of an SSB tax (61). Additionally, when these taxes are applied to diet drinks, the increase in price makes it harder for individuals to make healthy informed choices, as the non-diet versions may be more cost-effective to purchase.

While we understand that evidence for these newer and more controversial approaches is limited and needs further investigation, there is an opportunity for HCPs to move away from the historic ineffective approach of placing the onus on the individual, and instead help to create environments that are supportive of healthy lifestyles in a way that is easy, cost efficient, and which will become the norm. To enable healthcare providers to take a more holistic approach with patients through health education and promotion, they need to be supported by public health practices that improve access to care and tackle the obesogenic environment at its root. With this type of systemic change, we may find more success with obesity prevention and weight maintenance after initiating treatment, particularly when used in combination with medications, as we will discuss next.

### 2.2 Pharmacotherapy and surgery for weight loss in obesity

Due to the limited success in obesity management of lifestyle modification alone, medical treatment guidelines recommend use of adjunctive anti-obesity medication (AOM) for the best outcomes, including greater and more sustained weight loss (18, 19). An improved understanding of obesity pathophysiology and appetite regulation has led to the availability of a number of pharmacological treatments for obesity and overweight (62–64). AACE/ACE guideline-recommendations include orlistat, phentermine-topiramate and naltrexone-bupropion combinations, and the glucagon-like peptide-1 receptor agonist (GLP-1 RA) liraglutide (19). The US Food and Drug Administration (FDA) has also recently approved the GLP-1 RA semaglutide (2.4 mg subcutaneously once-weekly) and the GLP-1/glucose-dependent insuliotropic polypeptide RA tirzepatide (up to 15 mg subcutaneously once-weekly) as adjuncts to a diet and exercise for chronic weight management in adult patients with a BMI ≥27 kg/m^2^ and ≥1 weight-related complication, or a BMI ≥30 kg/m^2^ (65). Semaglutide has demonstrated efficacy and safety in patients with overweight or obesity, and is associated with a significant 11.8% body weight reduction compared with placebo (66–71). In patients with obesity, with and without type 2 diabetes, tirzepatide 15 mg was associated with a mean change in body weight of −14.7% and −20.9%, respectively (72, 73). Individuals with obesity may need to try multiple medications to find an optimal intervention, determined through conversations with their HCPs on health goals, comorbidities, responsiveness, side effects, willingness, and adherence challenges. Patient autonomy is a key practice, and patients should be encouraged to engage in a shared-decision making process with their clinician to choose the optimal obesity care for them as an individual (74).

Public health communication campaigns could play a role in public awareness and understanding in terms of what to discuss when considering pharmacotherapy for obesity management, including with providers and employers regarding insurance coverage. Many people have a perception that AOM therapy is for short-term use only (75, 76). However, discontinuation of AOM has been associated with a reduction in the achieved benefits, as demonstrated in the STEP 1 trial, wherein 1 year after withdrawing from semaglutide 2.4 mg and lifestyle intervention, individuals experienced weight recurrence of a mean of two-thirds of the weight lost after 68 weeks of treatment (77). This again highlights the chronic relapsing nature of obesity, and makes a case for life-long monitored adjunctive therapy with AOMs to prevent weight recurrence. Metabolic and bariatric surgery (MBS) is another effective option for patients with obesity across all BMI classes who have not achieved substantial weight loss with non-surgical interventions, with long-term data consistently demonstrating its safety, efficacy and durability, and decreased mortality compared with non-operative treatment methods (78). Additionally, public health campaigns could be used to expand acceptance of the use of AOMs and MBS. Weight bias and stigma can lead to an overvaluation of achievement of weight management success through diet and lifestyle interventions, such that we attribute virtue and assume a high level of self-control and discipline for the few that can achieve success with lifestyle intervention only. By default, this can result in the use of AOMs being rejected as an effective treatment option, as they may be perceived as a “crutch,” or “an easy way out,” suggesting a possible personal failing to achieve meaningful weight loss. In two studies of adults assigned to read about a woman who had lost 15% of her body weight either through diet and exercise or GLP-1 use, or bariatric surgery in one study, the results suggested greater beliefs that GLP-1 RA use and bariatric surgery were a weight loss “shortcut” (79, 80). Incorporating clinical trial results into public health campaigns may help to further educate people about the nature of obesity as a chronic relapsing disease with a physiological basis, and help to mitigate bias and stigma.

Despite the potential offered by AOMs, and the substantial evidence over the last decade for the efficacy and safety of MBS, both options are underutilized in the US, with most Medicaid programs not offering coverage for FDA-approved weight loss drugs (81) and only ~1% of individuals meeting guideline criteria actually undergoing MBS every year, despite most major insurance plans covering weight loss surgery (82–84). Out-of-pocket costs for AOMs can also create a health equity issue, with some GLP-1 RAs costing >$1,000 (85). Treatments may also have high copay amounts, which can be prohibitive for prospective patients. In one nationwide poll of 1,479 American adults (86), 54% of insured users noted difficulty with affording costs, and 23% answered that meeting costs was “very difficult.” Additionally, 57% of insured adults had to cover part of the cost themselves, and 19% had to pay the full cost themselves.

Improvements in US health insurance for coverage for AOMs and MBS could also positively impact employment and costs. One survey by the Obesity Action Coalition found that 44% of people with obesity would change jobs to gain coverage for treatment, and more than 50% would stay at a job they didn't like to keep coverage (87). Additionally, obesity has been shown to double the annual medical costs of adults relative to those of normal weight (a total of $5,010 per year vs. $2,504 (88)—costs that could potentially be offset by offering increased coverage. This feeds back into the potential for public health campaigns to increase public awareness of pharmacotherapy options, and understanding how and what to discuss with employers in terms of insurance coverage.

### 2.3 Multi-component interventions, including community-based approaches

We have already highlighted the importance of a multifaceted approach to obesity management with both lifestyle modification and adjunctive AOMs. Table 1 (89–95) provides an overview of community-based randomized trials and weight-loss programs in the US that have utilized such an approach, including personalized SMS messaging, counseling, support from community health workers, financial incentives, health technology, and health education (89–95). For all interventions listed, groups of people with access to more resources generally saw greater mean reductions in body weight, ranging from 0.6 to 4.0 kg. In a trial evaluating the effectiveness of behavioral weight loss maintenance, one group of participants who had maintenance sessions (practicing strategies associated with weight maintenance in the context of connecting faith with health) for 12 months following the initial 6-month behavioral treatment for weight loss, experienced a slower rate of weight recurrence compared to participants who did not have maintenance sessions (body weight change at 12 months: 0.90 kg vs. 1.58 kg, respectively; 93). While there are limitations to community-based approaches, including scalability, workforce availability and training, and insurance coverage, the strategies and interventions utilized are a useful starting point for informing policy, systems, and environmental (PSE) changes that need to be made by policy makers and stakeholders.

**Table 1:** Overview of community-based trials of obesity management in the US post-2015.

**Study**	**Geographic location and population**	**Design**	**Groups and obesity management strategy utilized**	**Weight loss outcomes**
Griffith et al. (89)	Atlanta, Georgia Middle-aged and older African American men	Community-based, cluster-randomized, longitudinal parallel group design; 4 churches	Control (*n* = 22) and weight loss (*n* = 21) Both conditions: • FitBit, Bluetooth-enabled scale, t-shirt, gift cards for participation, and 45-min small group physical activity led by a certified personal trainer Weight loss condition: • 45-min of health education, individually tailored SMS texts	Mean change in body weight: Control: −5.11 lbs (2.3 kg; *p* = 0.005) Weight loss: −7.14 lbs (3.2 kg; *p* = 0.005) Mean change in body fat: Control: −0.18 % points (*p* = 0.36) Weight loss: −2.37 % points (*p* = 0.005)
Ard et al. (90)	Deep South (Mississippi and Alabama) Rural African American women	Two-group cluster randomized controlled trial	Weight Loss Only (WLO; *n* = 154) and Weight Loss Plus (WLP; *n* = 255) Both groups: • Weight loss intervention sessions with a focus on moderate energy restriction and regular moderate-intensity physical activity, behavioral strategies, tailored reinforcement messages, continued self-monitoring, relapse prevention, social support and problem solving WLP: • Support for implementing strategies to promote healthy eating and/or physical activity in the local community e.g., community garden, enhancement of a walking trail, incentives for the purchase of a fresh produce from the local farmers' market, and a dance class	Mean change in body weight: WLO: −1.9 kg WLP: −2.7 kg
McVay et al. (91)	North Carolina Men and women with obesity and an obesity-related comorbidity	Randomized controlled trial	Participant-reported counseling (*n* = 141) and provider-documented counseling (*n* = 134); no counseling, general weight counseling or intervention-specific counseling Participant reported counseling: • General weight counseling: asked about their weight, advised them to lose weight, assessed their readiness to lose weight, referred them to a weight loss program, discussed weight loss medication, discussed weight loss surgery, or arranged a future contact to discuss weight • Intervention-specific counseling: talked to them about Track, asked about their progress toward Track goals, encouraged them to talk to their Track coach, encouraged them to take Track phone calls, talked to them about their weight change since starting Track, or provided ideas to help them meet Track goals Provider-documented weight counseling: • General weight counseling: patients' physical activity, patients' diet or eating behavior, specific weight loss goal, external weight loss program • Intervention-specific counseling: track was explicitly documented, track was referred to but not explicitly mentioned, provider reinforced engagement in Track, Track provider update was copied into visit notes	At 6–12 months: Patient-reported provider weight counseling: • Intervention specific counseling was associated with +0.4 kg (*p* = 0.68) compared to general weight counseling, and −0.6 kg (*p* = 0.60) compared to no counseling 0–12 months: Provider-documented weight coun-seling: • Intervention specific counseling was associated with −3.1 kg (*p* = 0.02) compared to general weight counseling, and −4.0 kg (*p* = 0.04) compared to no counseling
Lee et al. (92)	Southeast Texas Employees of the Pasadena Independent School District with overweight or obesity	6-month worksite-weight-loss program	Vibrant Lives Basic (VLB; *n* = 131), Vibrant Lives Plus (VLP; *n* = 87), and Vibrant Lives Plus + Support (VLP + S; *n* = 88) All groups: • Materials and tailored text messages VLP and VLP + S: • WIFI-enabled activity monitors and scales and participation in health challenges throughout the year VLP + S: • Coaching support	Mean change in body weight: VLB: −2.5 kg VLP: −2.5 kg VLP + S: −3.4 kg β = −2.34 SE = 0.45 *p* < 0.001
Yeary et al. (93)	Arkansas Delta Rural Blacks of faith	Cluster randomized controlled trial	Weight Loss Only (WLO; *n* = 218) and Weight Loss + Maintenance (WLM; *n* = 208)	
			WLO: • 6 months behavioral treatment for weight loss, followed by 12 months assessment WLM: • 6 months behavioral treatment for weight loss, followed by 12 months weight loss maintenance	Mean change in body weight from baseline to 6 months: WLO: −2.63 kg WLM: −2.45 kg *p* = 0.8045 Mean change in body weight from 6 months: 12 months: WLO: +1.04 kg (*p* = 0.0006) WLM: +0.48 kg (*p* = 0.1164) 18 months: WLO: +1.58 kg (*p* = 0.0002) WLM: +0.90 kg (*p* = 0.0408)
Rosas et al. (94)	San Mateo County, California Low-income Latinos with obesity and ≥1 heart disease risk factor	Community-based randomized controlled trial	Usual Care Control (UC; *n* = 41), Case Management (CM; *n* = 84), and Case Management + Community Health Worker (CM + CHW; *n* = 82) CM and CM + CHW: • 12-month intensive phase (12 group sessions + 4 individual sessions) followed by 12-month maintenance phase (3 group sessions + 1 individual session) CM + CHW: • 5 home visits in the intensive phase and 2 home visits in the maintenance phase	Mean body weight changes 6 months: UC: −0.9 kg CM: −1.6 kg CM + CHW: −2.1 kg CM + CHW vs. UC: *p* = 0.05 CM + CHW vs. CM: *p* = 0.65 12 months: UC: −0.7 kg CM: −1.4 kg CM + CHW: −1.9 kg CM + CHW vs. UC: *p* = 0.21 CM + CHW vs. CM: *p* = 0.76 24 months: UC: −0.6 kg CM: −1.0 kg CM + CHW: −1.0 kg CM + CHW vs. UC: *p* = 0.76 CM + CHW vs. CM: *p* = 0.98
Newton et al. (95)	Louisiana African American adults with obesity	Cluster-randomized trial design; 8 churches	Control (*n* = 29) and Intervention (*n* = 68) Control: • Encouraged to maintain normal eating and exercise habits for 6 months, and received 2–3 automated SMS messages per week on health topics specific to African American adults Intervention: • Community health coach leading 10 group sessions over 6 months, covering obesity management topics, and 5 automated SMS messages per week covering lesson content (*n* = 2), motivation (*n* = 1) and behavioral prompts (*n* = 2)	Mean change in body weight: Control: +0.2 kg Intervention: −1.4 kg *p* = 0.03 Effect size: 0.55 Mean percent weight loss: Control: +0.3% Intervention: −1.6% *p* = 0.04

Authors' review of PubMed searches relevant to obesity and weight loss. CM, case management; CM + CHW, case management + community health worker; SE, standard error; UC, usual care control; VLB, Vibrant Lives basic; VLP, Vibrant Lives plus; VLP + S, Vibrant Lives plus + support; WLM, weight loss + maintenance; WLO, weight loss only; WLP, weight loss plus.

**Figure 1:**
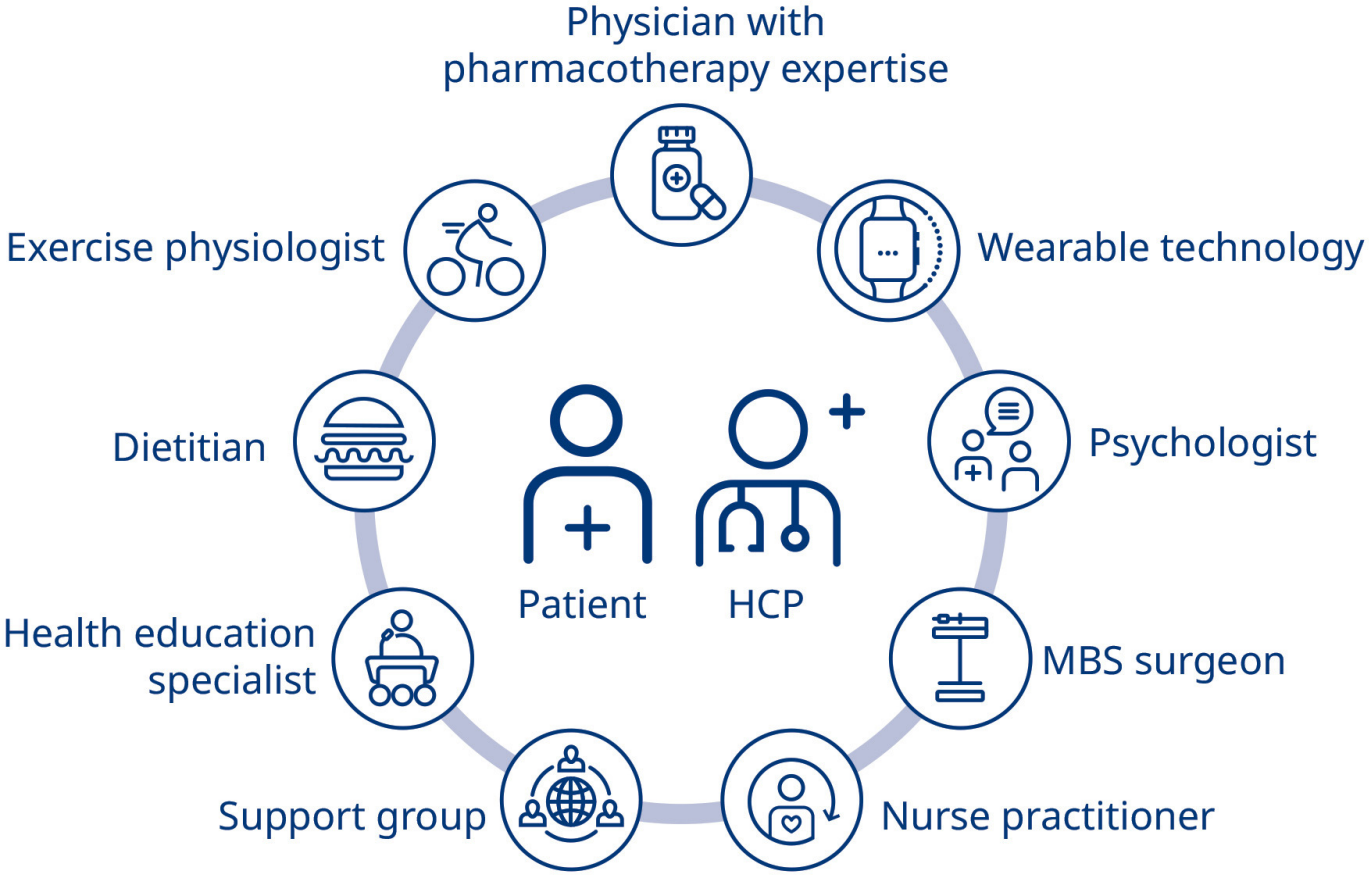
Integrated chronic care model (97). HCP, healthcare professional; MBS, metabolic and bariatric surgery.

The most effective way to implement a multifaceted approach to obesity management is through the adoption and utilization of an MDT, ideally including a physician with expertise in pharmacotherapy, a nurse and/or nurse practitioner, an exercise physiologist, a dietitian, a health education specialist, psychologist, and potentially a MBS surgeon (40, 96). Figure 1 (97) provides an overview of how a specialized integrated care model might address specific conditions or aspects related to chronic disease, including obesity, through employment of non-physician personnel. Several studies have demonstrated the effectiveness of an MDT in the primary care setting, including nurses, lay educators, dietitians, behavioral therapists, and health coaches (98–100). It would be beneficial to see further studies investigating the use of AOMs in conjunction with the input of an MDT on obesity outcomes, and particularly, weight management and recurrence. As obesity is a chronic relapsing disease, obesity management must also integrate aspects of personalized medicine, ensuring that patient circumstances, availability, costs and comorbidities inform treatment choices.

### 2.4 Existing public health initiatives

Healthy People 2030 is one of the existing public health initiatives for overweight and obesity management in the US. One objective, to reduce the proportion of adults with obesity, is currently not being achieved, with the proportion of adults with obesity continuing to increase (101). The evidence-based strategies backing this initiative are worksite programs (102), interventions including activity monitors (103), and technology-supported multicomponent coaching or counseling interventions to reduce weight (104) and maintain weight loss (105). It should be noted that many of these recommendations are based on outdated reviews of the literature, and while still helpful, it would be beneficial for an updated review to be carried out.

There are also numerous programs aimed at promoting healthy dietary habits and increasing physical activity to reduce obesity in adults, many of which use intervention approaches recommended by the Community Preventive Services Task Force (CPSTF). Two of these programs had weight change as a primary outcome [Table 2; (104, 106, 107)]. In ENERGY, the Experimental group (group-based behavioral intervention supplemented with telephone counseling and tailored newsletters) experienced significantly greater weight loss than the comparison group (weight management resources and materials and monthly telephone calls and/or emails) at 6, 12, and 18 months (106). Weight Loss for Life demonstrated significant weight loss in the Experimental treatment group (the Coach Approach exercise supported protocol, followed by group nutrition sessions), in both the weight loss phase and over the duration of the study (107). While both programs had significant results at ≥18 months, this is not long-term within weight management, and it is not known if the observed weight loss was maintained following the end of the programs. These programs and recommended intervention approaches reflect the continued focus of health officials on promoting diet and lifestyle changes; however, individuals will likely require a long-term follow-up plan and/or lifelong support to ensure that they continue with the recommended approaches and thus avoid relapsing disease. While the population-based programs reported in this section align with current clinical practice guidelines for lifestyle behavioral counseling in a community setting, it is important to integrate these with healthcare-based interventions for individuals and groups, along with ecological approaches to foster supportive, healthy environments.

**Table 2:** Existing obesity evidence-based programs in the US.

**Program title and year**	**Geographic location**	**Purpose**	**CPSTF recommendation utilized**	**Intended audience**	**Design**	**Key findings**
Exercise and Nutrition to Enhance Recovery and Good Health for You (ENERGY), (106)	San Diego, California; Denver, Colorado; St Louis, Missouri; Birmingham, Alabama	Promote healthy dietary habits to reduce obesity	Technology-supported multicomponent coaching or counseling to reduce weight (104)	Female breast cancer survivors aged ≥21 years with overweight or obesity	4-year randomized clinical trial Control and Intervention groups Control (*n* = 348): • Weight management resources and materials, monthly telephone calls and/or emails Intervention (*n* = 344): • Group based behavioral intervention, supplemented with telephone counseling and tailored newsletters	*Mean weight change vs. baseline at 6 months:* Control: −1.2 kg Intervention: −5.0 kg *p* = 0.002 *Mean weight change vs. baseline at 12 months:* Control: −1.2 kg Intervention: −5.3 kg *p* = 0.003 *Mean weight change vs. baseline at 18 months:* Control: −1.2 kg Intervention: −4.4 kg *p* = 0.02
Weight Loss for Life, (107)	Eastern US	Promote healthy dietary habits and increase physical activity to reduce obesity	N/A	Women aged ≥21 years with obesity and a self-reported goal of weight loss	Comparison (COM) and Experimental (EXP) treatment COM (*n* = 55): • Print manual plus telephone follow-ups EXP (*n* = 55): • The Coach Approach exercise-support protocol for 2 months, followed by group nutrition sessions with a focus on generalizing self-regulatory skills from an exercise support to a controlled eating context	*Weight loss phase (6 months) mean weight reductions:* COM: −2.09 kg; *p* < 0.001 EXP: −5.73 kg; *p* < 0.001 *Full study duration (24 months) mean weight reduction:* COM: −1.25 kg; *p* = 0.068 EXP: −5.11 kg; *p* < 0.001

National Cancer Institute Obesity Evidence-Based Programs Listing. Only trials where weight loss was a primary outcome have been reported. COM, comparison; CPSTF, Community Preventive Services Task Force; EXP, experimental; N/A, not applicable.

To date, there are only 3 CDC-funded state and local programs working to increase healthy eating and active living, and to prevent adult and childhood obesity (108). There is inadequate funding to sufficiently support every state, with respect to effective community prevention programs (39), with only $0.31 per person allocated for CDC obesity-prevention efforts (109). There are large differences in the funding awarded to CDC programs, with the Division of Diabetes Translation having a 2022 budget of $193.4 million (110), compared with the 2021 budget for the Division of Nutrition, Physical Activity, and Obesity of $56.9 million (111). This may highlight inequities at a public health level of how importantly obesity prevention and management is viewed compared with other diseases, and could also reflect that obesity is still not properly recognized as a chronic condition, requiring multiple approaches and lifelong treatment.

This also relates back to the relationship between obesity and the risk of developing complications of obesity, as discussed earlier. Obesity drives the development of many chronic diseases, including T2D. Effective resource allocation to improve multidisciplinary team engagement to address obesity early could mitigate or eliminate the development of these complications, thus being an effective primary prevention strategy for many chronic diseases like T2D that increase morbidity and mortality and disproportionately affect individuals from marginalized and low-resource communities. Addressing obesity as a driver of other diseases may also be another strategy to reduce stigma and increase access to care that addresses disparities and preventable disease. However, other critical problems must be considered in this context, such as perceptions about the relative roles of public health and healthcare in obesity, the relative lack of data on the effectiveness of the strategies historically applied, and a possible lack of political willpower for effective intervention at public health level.

Public health awareness and advocacy campaigns nevertheless play a key part in educating people that obesity is a chronic disease, and addressing historical misinformation and stigma around weight. “Obecity, USA” is a public health advocacy campaign that aims to both raise awareness and shift public perception of obesity as a disease, and provides individuals and communities with evidence-based research and resources essential to leading healthy lives (112). The Obesity Action Coalition has also developed several public health campaigns regarding obesity management including “Your Weight Matters” (113), “Obesity Care Week” (114), and “Stop Weight Bias” (115). Unlike other public health campaigns, Obesity Care Week provides resources not just for people with obesity, but also for HCPs. As HCPs are generally under-prepared to tackle obesity management, such resources are important for improving relationships between people with obesity and their primary care providers, and enabling more open and productive conversations regarding obesity management.

Current public health initiatives are still largely focused on the actions of the individual and making changes to diet and lifestyle, and supported by the above-mentioned CDC programs targeting obesity prevention and mitigation (108). There is some evidence that a significant proportion of the population have already begun to adopt lifestyle changes. Although not directly comparable due to changes in scoring systems and age categories, HEI scores have generally increased from ~54 for adults in 2005–2006 (116), to 57 in 2017–2018 (117). While highly personalized approaches such as diet and exercise modification must consider individual lifestyle choices, effective design of public health initiatives must also consider each respective community; the unique needs, resources and risks of varying populations must be addressed going forward. Assessment of the unique social, biological, and behavioral needs and preferences at both the individual and population levels is central to targeting strategies for the given circumstances (i.e., individual and small groups or entire communities); such factors may dictate which public health interventions will be more relevant and successful in different areas and for different populations. However, future public health initiatives need to do more to encompass, advertise, and improve access to personalized lifelong obesity management options, including utilization of AOMs and MBS.

### 2.5 Additional barriers to accessing obesity treatment

Some of the barriers to obesity prevention and management already discussed are an obesogenic environment, insufficient access to AOMs (due to factors such as arbitrary insurance coverage decisions or prohibitively high co-pay requirements) and underutilization of AOMs and MBS, bias and stigma in the context of obesity, and related factors e.g., use of AOMs, underutilization of MDTs, and lack of funding.

In relation to public health campaigns, while they can improve awareness and education among the general public, there is the potential that they can create an inadvertent additional barrier in that obesity-related public health media campaigns could be perceived as stigmatizing rather than motivating. A review of major obesity public health campaigns in the US, UK, and Australia found that messages perceived to be the most motivating and positive did not mention the word “obesity,” with the focus instead on making healthy behavioral changes, and no reference to body weight. Conversely, the messages with the lowest intentions to comply and the most negative ratings were those that had been publicly criticized for their stigmatizing content (118). Another study found that the use of stereotypical images in anti-obesity campaigns were rated as the most stigmatizing, and were also associated with more desired social distance from the target, and lower positive and higher negative trait ratings of the target, compared to counter-stereotypical images, neutral images, or no images (119).

Although HCPs are increasingly recognizing obesity as a chronic disease, it is often not treated as such and this may relate to a lack of education among both HCPs and people with obesity. A thematic analysis revealed weight bias and negative views and attitudes among HCPs with regards to patients with obesity, their role in obesity assessment, and inadequate training and equipment for obesity assessments, along with citing a lack of time, lack of incentives in the health system, and increased financial cost implications (120). The lack of comprehensive training could be reflective of a legacy of low priority given to obesity education in US medical schools, with only 10% of students in a survey responding that they were “very prepared” to manage patients with obesity, and 30% of schools reporting little or no education in nutrition and behavioral interventions, appropriate communication with patients with obesity, or pharmacotherapy (121). A cross-sectional study of HCPs established that around one-third did not have sufficient knowledge of how to diagnose or treat obesity (122). If HCPs are unable to diagnose obesity, this limits referral to appropriate treatment resources, such as an MDT. The lack of knowledge of obesity amongst HCPs may contribute to knowledge gaps among patients with respect to obesity as a disease, their weight status and how much it is likely to affect their future health, and available treatment options (43). Improving health education, among both HCPs and patients, is key to overcoming some of the barriers to obesity management, including weight bias and stigma. One study found that HCPs who received a 4-h continuing medical education (CME) intervention containing theory-based elements of changing attributions of responsibility of obesity, increasing empathy, creating self-awareness of weight bias, and creating a bias-free culture, had significantly reduced self-reported negative obesity stereotypes compared to baseline (*p* < 0.001), and significantly increased self-reported empathy (*p* = 0.006) and confidence in caring for patients with obesity (*p* < 0.001) immediately post-intervention, which were maintained at 4- and 12-month follow-up. Additionally, compared to HCPs who did not attend the CME intervention, those who did had significantly increased odds (range 60%−212%) of both diagnosis and obesity-related referrals in the 12 months following the intervention (123). It would also be beneficial for professional training of HCPs to focus on methods for assessing the risks of overweight and obesity and associated comorbidities, individual to the patient, so that appropriate referrals can be made to clinical and public health programs and resources, whereby individual treatment plans can be developed using a combination of lifestyle behavioral assessment, clinical risk assessment data, and personal preference.

With respect to expanding access to obesity care in the primary care setting, this links back to an MDT approach in which team-based care could be utilized for lifestyle-focused treatment. There are challenges to sustainably funding and staffing a primary care-based weight management program, although one potential solution would be to use chronic care management codes for patients with obesity (124). This would require an overhaul of diagnostic coding, medical billing, and reimbursement practices in the US. Research by Avalere, a healthcare consulting firm, identified 5 key diagnostic coding and administrative barriers to care for people with obesity: (1) overall lack of understanding of appropriate coding for obesity contributes to underdiagnosis at all stages of a patient's care journey; (2) treatments for complications associated with obesity are often prioritized over coding for and treating obesity; (3) system challenges deter providers from utilizing obesity-specific diagnosis codes, resulting in underutilization; (4) low utilization of preventative care and chronic care management programs to treat people with obesity exists due to coverage burden or associated hurdles and; (5) provider challenges in diagnosing and coding patients for obesity can create barriers to shared decision making (125). As of October 1, 2024, new ICD-10-CM diagnosis codes have been introduced for adult and childhood obesity, which align with the newest recommendations from professional societies. These will help to improve accuracy of obesity severity, improve coding practice as obesity is currently under-coded, enhance the usefulness of data, and help reduce stigma and bias by using accurate and clinically relevant terms (126). Although more still needs to be done, this is an important first step toward improving diagnosis and diagnostic coding of obesity, and therefore appropriate treatment referrals.

### 2.6 What changes need to be made?

Obesity can be described as a systems problem, whereby it is the result of multiple interconnected components, which interact in complex ways (127). This is evidenced by several factors, including global scope, heterogeneous patterns of prevalence, wide-ranging impacts, lack of a single cause, and the failure of a single solution (127); thereby a systems integrated approach to obesity prevention and management is likely to be required.

**Figure 2:**
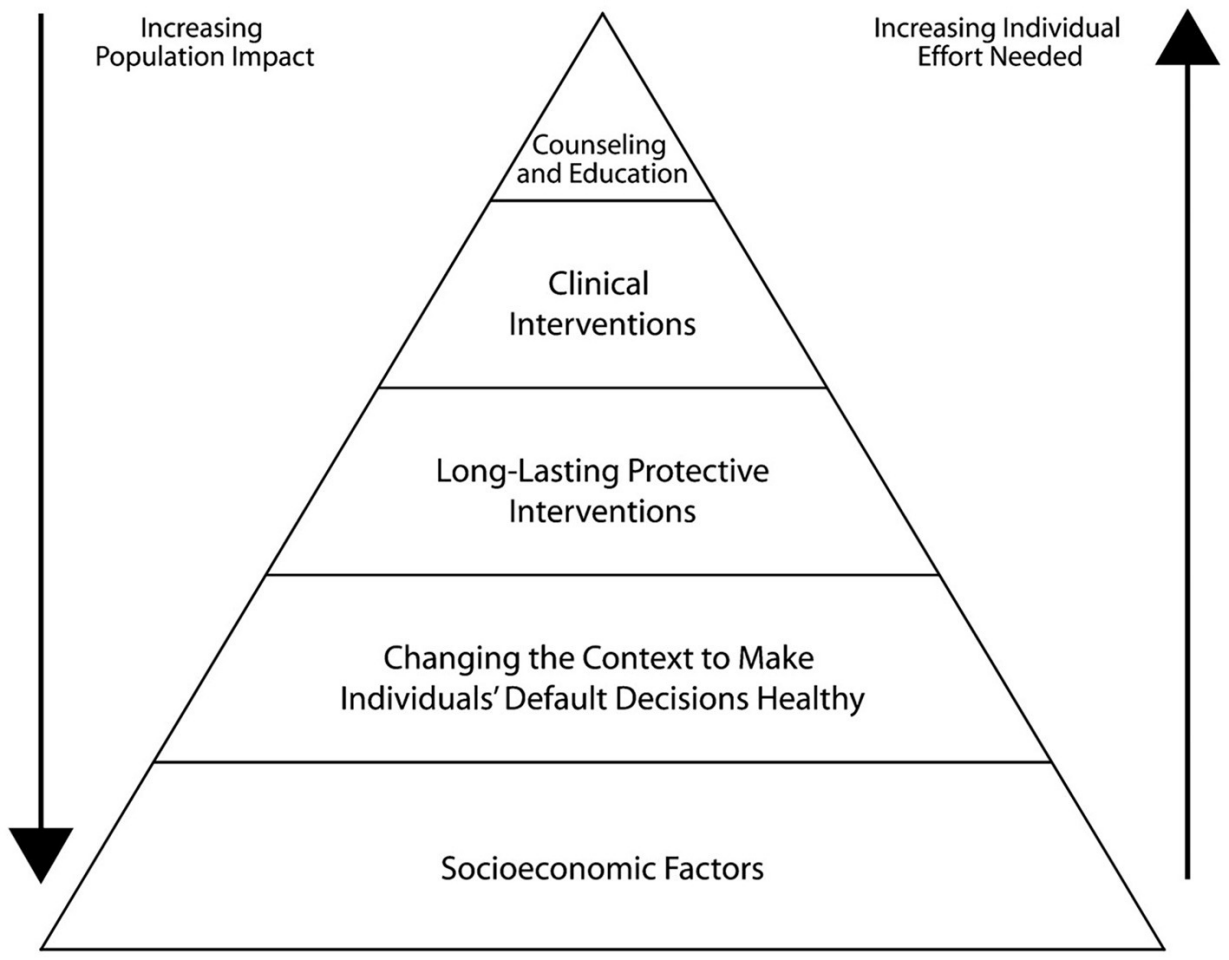
Health impact pyramid. Reproduced with permission from Frieden (128). AOM, anti-obesity medication; MBS, metabolic and bariatric surgery; SSB, sugar-sweetened beverage.

The 5-tier health impact pyramid is an alternative conceptual framework to improve health through different types of public health interventions. It proposes that the bottom two tiers, comprised of socioeconomic factors, or social determinants of health (SDOH), and changing the context to make individuals' default decisions healthy, generally improve health for more people at lower unit cost than those levels at the top of the health impact pyramid [long-lasting protective interventions, clinical interventions, and counseling and education; (128); Figure 2]. With respect to unit cost, this refers to the assumed cost per individual. Within the health impact pyramid, factors closer to the base of the pyramid have greater population impact, whereas the factors further from the base are designed to help individuals, and so while broad changes at a population level may be more expensive, the overall cost per individual is lower than changes implemented at the top tiers of the pyramid. PSE change is the key way in which the first two levels of the health impact pyramid can be addressed, bringing about much needed improvements in infrastructure, funding, and resources that are needed by public health officials to have the biggest impact and population reach. As suggested by TFAH, these can include measures such as increasing funding for obesity prevention programs, supporting multisector collaborations to address upstream drivers of chronic disease, instituting economic policies to reduce poverty, and ensuring that health equity in planning and decision-making at federal agencies is prioritized (39).

There are some existing frameworks that aim to inform local and state action for obesity prevention, with respect to PSE approaches. The National Council on Aging identified 10 policy solutions for improving obesity care in older adults, addressing a multitude of challenges, and recognized that action needs to be taken by a variety of people and groups, including researchers, healthcare providers, public health professionals, policymakers, and other stakeholders (129). These policy solutions include personalizing obesity treatment, considering several measures of health (i.e., not just BMI), accounting for all conditions that affect health and weight (e.g., SDOH), and better coordination between health systems and community-based weight management and obesity treatment services (130). These are again similar to the proposed recommendations from the TFAH, and at local and state governance levels include addressing food production and processing, distribution, marketing, retail, restaurants and food service, infrastructure and planning, education, employment, transportation, sports and recreation.

More also needs to be done to foster better partnerships between public health system organizations and healthcare settings, to address the five domains of SDOH [economic stability, education access and quality, health care access and quality, neighborhood and built environment, and social and community context; (131)], and to close disparities and gaps in clinic-to-community settings. With respect to economic stability and access to healthcare, this could be reflected in policies designed to reduce poverty, expand access to healthcare coverage and opportunities for coordination between public health and healthcare coordination, addressing root causes of health disparities, and prioritizing SDOH strategies. Policies for addressing neighborhood and built environment have been discussed earlier in this article in the context of tackling the obesogenic environment, with improving food security also relating to social and community context. Community-focused interventions can also feed into these policies, such as Medicaid reimbursement for interventions such as “Food is Medicine” (132), which includes produce prescriptions. On the whole, future public health policies and approaches need to be geographically adaptable, in order to address the specific needs of individual communities. People with obesity living in rural areas are more likely to be affected by issues such as limited access to healthy foods, health services, and exercise facilities, whereas those living in urban areas are more likely to be affected by issues such as neighborhood safety (133, 134), and, in turn, these factors will dictate which public health changes will be more relevant and successful in different areas and for different populations.

We recognize that policy change is just as complex as healthcare change, and one of the key components of this is that public health officials do not have the sole authority to tackle crises, nor the input and infrastructure, with only 5% of the $4.5 trillion spent on health in 2022 in the US going toward targeted public health activities (135). Implementation of effective obesity-prevention policies based on recommendations from national and international organizations has been slow and inconsistent, and a report from *The Lancet* has recognized this by the collective term “policy inertia – the combined effects of inadequate political leadership and governance to enact policies, strong opposition to those policies by powerful commercial interests, and a scarcity of demand for policy action by the public” (136).

As demonstrated in Tables 1 and 2, innovative, evidence-based interventions have not been fully identified or systematically applied in public health to date. This is a crucial area for future research, in order for public health campaigns and interventions to have a scientific basis, with proven results. Besides funding clinical trials to evaluate the effects of drugs regulated by the FDA, the National Institutes of Health (NIH) also awards funds to clinical trials of other interventions that are not FDA-regulated (137). This could be a key area for stakeholders and policymakers to target in the future with respect to clinical trials investigating an MDT approach to obesity management and treatment.

## 3 Conclusion

It is clear from the evidence that a holistic integrated approach to obesity management results in better outcomes for people with obesity as it is a chronic, complex, and often relapsing disease. Existing measures and public health initiatives have limited reach, and, despite a long history of limited effectiveness, still have a predominant focus on the action of individuals. More work is needed to advocate the importance of changing strategies for obesity management to public health officials and enable them to adapt their strategies to take advantage of the new science on obesity e.g., campaigns fighting stigma, messaging encouraging the public to consider all levels of intervention, policy advocacy for AOM coverage, and environmental change. PSE changes at the national, state, county, and city levels are key to tackling many of the barriers to obesity prevention and treatment, particularly with regard to making changes to SDOH; this can be achieved through increasing and improving public health funding and infrastructure for obesity prevention and management, which are currently insufficient. For the time being, existing models and recommendations identified in current clinical practice health guidelines, such as the AAC/AHA/TOS, AACE/ACE, and TFAH, have a strong foundation with sufficient evidence to guide clinical and public health strategies that can be refined in the future as further solutions are identified. Public health officials, stakeholders and policymakers must recognize that obesity is chronic disease that needs to be addressed now, for the sake of the US population and future generations.
